# Do we need a theory-based assessment of consciousness in the field of disorders of consciousness?

**DOI:** 10.3389/fnhum.2014.00402

**Published:** 2014-06-04

**Authors:** Alexander A. Fingelkurts, Andrew A. Fingelkurts, Sergio Bagnato, Cristina Boccagni, Giuseppe Galardi

**Affiliations:** ^1^Research Department, BM-Science – Brain and Mind Technologies Research CentreEspoo, Finland; ^2^Neurorehabilitation Unit, Rehabilitation Department, Fondazione Istituto “San Raffaele-G. Giglio,”Cefalù, Italy; ^3^Neurophysiology Unit, Rehabilitation Department, Fondazione Istituto “San Raffaele-G. Giglio,”Cefalù, Italy

**Keywords:** (Un)consciousness, disorders of consciousness (DOC), vigilance, levels of consciousness, electroencephalogram (EEG), vegetative state (VS), minimally conscious state (MCS)

Adequate assessment of (un)consciousness is not only of theoretical interest but also has a practical and ethical importance, especially when it comes to disorders of consciousness (DOC). Accurately determining the presence or absence of consciousness in patients with DOC allows informed decisions to be made about long-term care support, referral for rehabilitation, pain management and withdrawal of life support.

In spite of significant progress in neuroimaging and the introduction of clear-cut clinical diagnostic criteria, determining the (un)consciousness still presents an important clinical problem: errors are common and have been shown to be as high as 37–43% (Tresch et al., 1991; Childs et al., 1993; Andrews et al., 1996; Schnakers et al., 2006).

Assessment errors arise because the evaluation of patients with DOC is based mostly on clinical observation of subjectively interpreted behavioral responses, while conscious experience often occurs without any behavioral signs. Additionally behavioral responses of such patients are usually limited by their cognitive dysfunctions and/or by their frequent motor impairment. Therefore, determining if a non-communicative or minimally communicative patient is phenomenally conscious poses a major clinical and ethical challenge. For this reason, there is a need for paraclinical diagnostic markers for the presence or absence of consciousness.

We believe that a theoretical account of what conscious experience is and how it emerges within the brain will advance the search for appropriate neuromarkers of the presence or absence of consciousness in non-communicative brain-damaged patients.

In our view, several important considerations need to be kept in mind:

## Consciousness vs. vigilance

Consciousness is often conceptualized as a phenomenon with two components: wakefulness and awareness (Posner et al., 2007). Though such understanding is currently quite wide-spread, it confuses and mixes two different and independent phenomena: subjective awareness and vigilance. While awareness is an important component of consciousness, wakefulness belongs to the vigilance domain. Independence of these two concepts can be demonstrated by examples from a daily life: (a) we are able to unconsciously perform complex actions like brushing our teeth or driving a car while being completely awake; (b) being at the same level of wakefulness we are usually aware of some events/stimuli while unaware of others; and (c) during sleep we can be aware of our phenomenal experience (dreams) but are not awake. Hence, wakefulness is not a component of consciousness but of vigilance. Vigilance, however, affects consciousness by limiting the amount of information available for conscious access (Rusalova, 2006), thus affecting the amount of content (Overgaard and Overgaard, 2010).

## Is consciousness gradually continuous or discrete (“all-or-none”)?

From the abovementioned fallacy, another misconception arises—levels of consciousness. The assumption is that consciousness itself can be somehow diminished (less consciousness) or increased (more consciousness), and thus considered to be gradual (Laureys et al., 2002; Vanhaudenhuyse et al., 2010a). However, there is no introspective evidence to support this widely accepted idea (Overgaard and Overgaard, 2010). Indeed, from a third-person perspective, consciousness presents itself in varying amounts, depending on the level of vigilance of the studied subject. However, what is important is that from the first-person perspective one is either discretely fully aware or unaware of something. It is the amount of content that varies gradually (Overgaard and Overgaard, 2010). There is no additional degree of consciousness during such awareness of the content (for a discussion see Fingelkurts et al., 2012a). In other words, consciousness is not merely a quantitative matter of a degree but in fact a qualitative matter of absence or presence of a particular state (Plum et al., 1998). In this sense, when consciousness is separated from arousal/wakefulness, then it is more of a categorical (all-or-none) phenomenon rather than a continuous (gradual) one (Fingelkurts et al., 2012a). It is the degree of vigilance (wakefulness) that conflates the expression of consciousness, resulting in an illusion of its continuous or graded nature (Hudetz, 2010).

## What is then consciousness?

**Figure 1:**
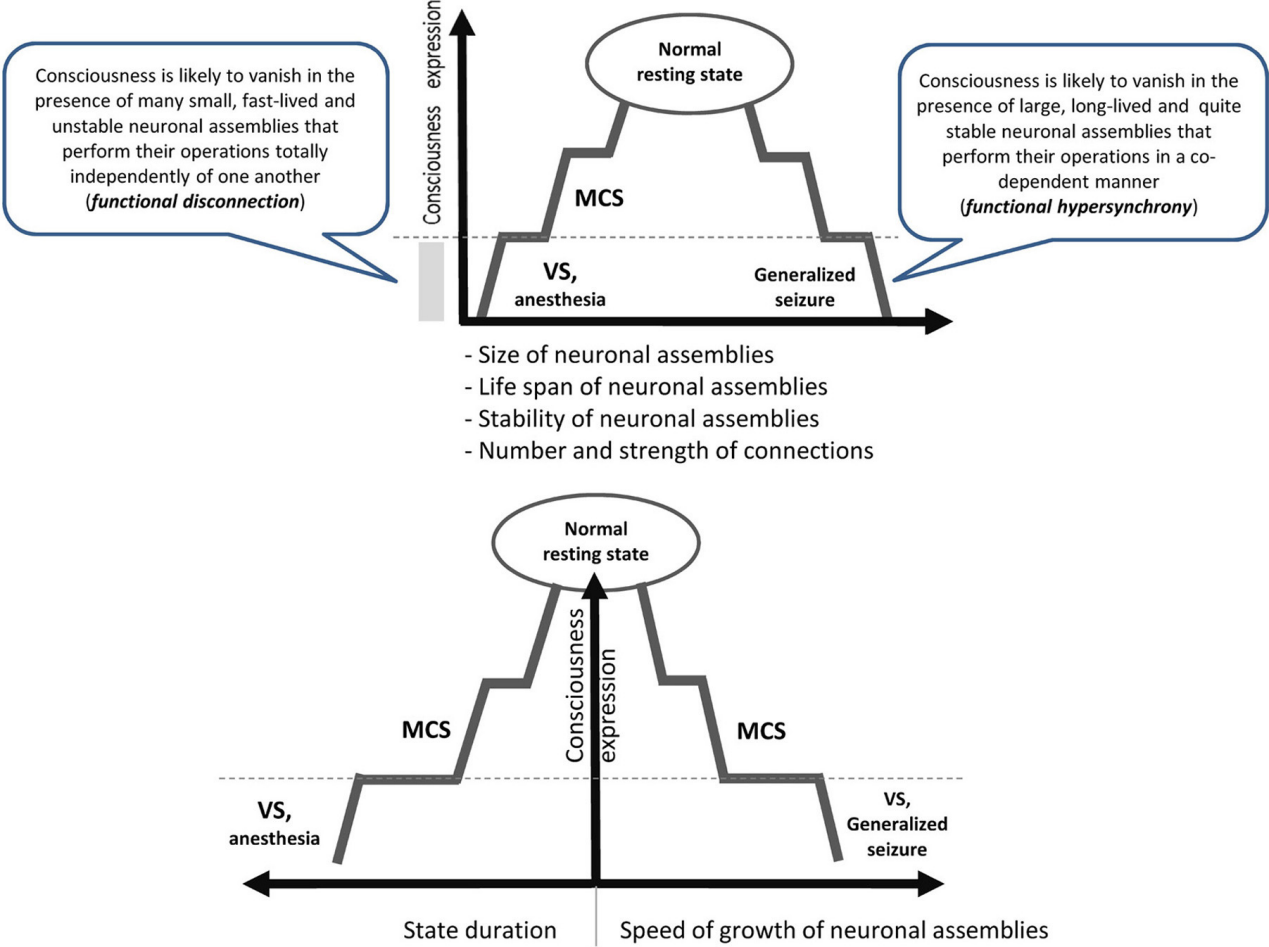
**Schematic illustration relating consciousness expression and neuronal assembly characteristics**. The stepwise line represents the idea that gradual changes in neuronal mechanisms need to be accumulated to reach a particular threshold level required for qualitative change in the functional state (Bagnato et al., 2013). During VS as a result of a brain injury, the functions of the neural net subtending consciousness (awareness) are reduced in both hemispheres below the threshold level required for minimal consciousness expression. The recovery of consciousness is a dynamic process that involves many plastic changes in many brain structures. If this reorganization crosses the threshold of the minimal neuronal mechanisms that are jointly sufficient for any conscious awareness (particular level of the size, life-span, stability and speed of growth of neuronal assemblies, as well as the amount and strength of functional connectivity between them), the patient will regain consciousness (Fingelkurts et al., 2012a,b). The critical factor regulating the occurrence or absence of consciousness recovery is the distance of these functional characteristics of neuronal assemblies from this threshold level (Bagnato et al., 2013).VS, vegetative state; MCS, minimally conscious state; dashed horizontal line illustrates a threshold of the minimal neuronal mechanisms that are jointly sufficient for any conscious awareness to emerge.

It is reasonable to assume that to be conscious is to be in a particular state which has projections onto mental/ psychological, neurophysiological and cognitive/behavioral dimensions (Edelman, 1989; Sokolov, 1990; Flohr, 1991; Tononi, 2008). Currently we do not know all parameters of this state, but recent empirical studies have provided several important observations (see Figure 1):

- The realization of a particular state of consciousness requires particular level of vigilance [and hence a preservation of the autonomic nervous system (Plum and Posner, 1980; Wijnen et al., 2006)], a corresponding functional state and physical integrity of the brain (Pistoia and Sara, 2012).
- A given conscious state should have a particular duration: it must be longer than the time it takes for the simplest cognitive act to be completed, which is on the order of several hundreds of milliseconds (Pöppel, 1997; Geissler et al., 1999; VanRullen and Koch, 2003). It seems that duration less than this threshold makes a state un-conscious (still mental domain) or non-conscious (non-mental neurophysiological domain) (for a discussion see Fingelkurts et al., 2010; Bagnato et al., 2013).
- It seems that the state of consciousness is supported by medium values of such characteristics of neuronal assembles as their functional size, life span and stability (Figure 1). Indeed, consciousness is lost when neuronal assembles' size, life span and stability decrease as is the case for patients in vegetative state (VS) or under general anesthesia (Greenfield and Collins, 2005; Fingelkurts et al., 2012b). Likewise, consciousness is lost when these characteristics are changed in the opposite direction as in the case of patients during the generalized seizure (Martin, 1991). Curiously, the speed of the growth of neuronal assemblies is slow during a conscious state but it increases significantly when consciousness is lost (Fingelkurts et al., 2012b).
- Similarly, both low (like in VS or general anesthesia) and high (like in seizure) levels of synchrony among neuronal assemblies result in a dramatic loss of consciousness (Flohr, 1995; Mashour, 2004; John and Prichep, 2005; Blumenfeld, 2008; Cavanna and Monaco, 2009; Hudetz, 2010; Fingelkurts et al., 2012b) (Figure 1).
- High strength of default mode network synchrony is required to support representational content integrated within the first-person perspective (Vanhaudenhuyse et al., 2010b; Fingelkurts et al., 2012c).
- The state of consciousness is dominated by EEG fast-alpha and beta oscillations (Fingelkurts et al., 2012a,b,c see also Gugino et al., 2001; Kuizenga et al., 2001; Babiloni et al., 2006, 2009; Rusalova, 2006; Başar and Güntekin, 2009) that may be considered necessary and a minimally sufficient neural condition for a conciseness to be expressed.
- The state of consciousness is independent from specialized cognitive processes like episodic memory, language, introspection or reflection, sense of space, sense of body, sense of self, or sensorimotor processing, or attention as it follows from neurological evidence (for the reviews see Tononi and Laureys, 2008; Boly et al., 2009).

Taken together (Figure 1) these findings suggest that consciousness is an emergent phenomenon of coherent dynamic binding of multiple, relatively large, long-lived and stable, but transient alpha and beta generated neuronal assemblies organized as synchronized patterns within a nested, hierarchical brain architecture. It seems that these are minimally sufficient conditions at the more basic level (brain) that are required for the emergent quality (conscious mind) to manifests itself. Indeed, if phenomenal consciousness is a biological phenomenon within the confines of the brain, then there must be a specific level of brain organization and a specific spatial–temporal grain in it where consciousness resides. In other words, we could expect that at the lower (in comparison with the phenomenal consciousness) level of brain organization there should be non-experiential entities (some complex electrophysiological mechanisms) that function as the direct realization base of the phenomenal world (Fingelkurts et al., 2010, 2013a). The abovementioned nested hierarchical architecture of separate and synchronized neuronal assemblies forms the very particular level of brain functioning, so-called operational architectonics level, which on the one hand intervenes between physical level of the brain where it literally resides, and on the other, is isomorphic to the experiential/subjective phenomenal structure of the mind (Fingelkurts et al., 2010). In other words, the level of the operational architectonics has emergent properties relatively independent from the neurophysiological/neuroanatomical properties of the physical level. And the phenomenal level supervenes on this operational level with one-to-one correspondence thus making it ontologically inseparable from it (though it is separable from the brain neuroanatomical processes through the operational level) (Fingelkurts et al., 2013a).

## Analytic model for assessing consciousness

Patients in VS or in minimally conscious state (MCS) offer a unique opportunity to study the neural basis of (un)consciousness due to the fact that impairment in awareness of self and environment is dissociated in such patients from preserved and stable wakefulness. We believe that an appropriate level of consciousness description should articulate the operational level of brain organization where the phenomenal/conscious phenomena reside (Fingelkurts et al., 2013b). Electroencephalogram (EEG) is a suitable and adequate measure for the instrumental analysis of such operational level, because it (a) provides a direct (in contrast to indirect fMRI an PET) measure of the behavior of large-scale neuronal networks with a millisecond temporal resolution and reflects functional properties and states of brain functioning as well as being closely connected to information processing in/among neuronal assemblies (for a discussion see Fingelkurts et al., 2012a) and (b) enables clinicians to assess spontaneous brain activity at each level of vigilance and in any state of consciousness, bypassing the need to elicit a behavioral or any other response from the patient (Vanhaudenhuyse et al., 2010b).

Following Baars's (1988) recommendation, an experimental analytic model for the assessment of consciousness should consider only those EEG parameters that satisfy the rule: (i) NORM ≥ MCS > VS for subjective awareness of self and environment, (ii) NORM ≥ MCS < VS for subjective unawareness of self and environment. This model was already successfully used in several recent studies (Fingelkurts et al., 2012a,b,c, 2013b).

In conclusion we argue that in the situation where there is no consensus on what would constitute the reliable markers of consciousness in the absence of the subject's report, a theory-based insight into neural substrates and mechanisms involved in conscious content may be useful for detecting the presence of conscious experiences in non-communicating subjects.

## Do we need a theory-based assessment of consciousness for proper rehabilitation of patients with DOC?

On the basis of the foregoing concepts, we may assume that patients with similar clinical behavior (i.e., VS or MCS) differ considerably in their level of operational architectonic dysfunction and that in turn translates into different expression of consciousness (Fingelkurts et al., 2012b). This is a critical point, if we consider that chances of recovery from a DOC (particularly, from a VS) depend on the interaction of two main factors: (i) the degree of impairment of neuronal systems supporting consciousness, and (ii) the amount of spontaneous and rehabilitation-induced plastic changes aimed to restore brain functions and connectivity within nested operational architectonics (Bagnato et al., 2013). If so, the precise measurement of brain dysfunction characteristics will be decisive, as it will allow rehabilitative treatments to be tailored for each patient. In the future, we may test the effectiveness of specific interventions (i.e., cognitive rehabilitations, drugs or neurostimulation) in patients in VS or MCS by evaluating the effects of the treatments on the patients' neuronal assembly characteristics mentioned earlier. We will then be able to choose the best rehabilitative intervention (or a suitable combination of treatments) for each patient with severe DOC by taking in consideration neurophysiological markers that are easily quantifiable at any stage of rehabilitation.

### Conflict of interest statement

The authors declare that the research was conducted in the absence of any commercial or financial relationships that could be construed as a potential conflict of interest.
